# Neuronally derived extracellular vesicles: an emerging tool for understanding Alzheimer’s disease

**DOI:** 10.1186/s13024-019-0317-5

**Published:** 2019-06-10

**Authors:** Luke S. Watson, Eric D. Hamlett, Tyler D. Stone, Catrina Sims-Robinson

**Affiliations:** 1 Department of Neurology, Medical University of South Carolina, 96 Jonathan Lucas Street, 301 Clinical Sciences Building, MSC 606, Charleston, SC 29425 USA; 2 Molecular and Cellular Biology and Pathobiology Program, Medical University of South Carolina, Charleston, Charleston, SC 29425 USA; 3 Department of Pathology and Laboratory Medicine, Medical University of South Carolina, Charleston, SC 29425 USA; 4 Honors College, College of Charleston, Charleston, SC 29424 USA

**Keywords:** Autophagy, Beta-amyloid, Cognitive impairment, Dementia, Exosome, Mammalian target of rapamycin, Neurodegeneration, Tau

## Abstract

In order for Alzheimer’s disease (AD) to manifest, cells must communicate “pathogenic material” such as proteins, signaling molecules, or genetic material to ensue disease propagation. Small extracellular vesicles are produced via the endocytic pathways and released by nearly all cell types, including neurons. Due to their intrinsic interrelationship with endocytic processes and autophagy, there has been increased interest in studying the role of these neuronally-derived extracellular vesicles (NDEVs) in the propagation of AD. Pathologic cargo associated with AD have been found in a number of studies, and NDEVs have been shown to induce pathogenesis in vivo and in vitro. Exogenous NDEVs are also shown to reduce plaque burden in AD models. Thus, the NDEV has the potential to become a useful biomarker, a pathologic potentiator, and a therapeutic opportunity. While the field of NDEV research in AD is still in its infancy, we review the current literature supporting these three claims.

**Electronic supplementary material:**

The online version of this article (10.1186/s13024-019-0317-5) contains supplementary material, which is available to authorized users.

## Background

For decades, therapies for Alzheimer’s disease (AD) have targeted beta-amyloid (Aβ) and phosphorylated tau proteopathies. Unfortunately, research has not yielded any viable therapeutics over the past 20 years. This lack of progress may be due to the complexity and heterogeneous nature of clinical AD. Approximately 80% of AD subjects present with multiple pathologies *post-mortem* such as amyloid, tau, vascular disease, Lewy Bodies, and alpha-synuclein [1–3]. Interestingly, nearly 70% of cognitively normal individuals also present with the same *post-mortem* pathology. Hence, there is a need for a paradigm shift in the field away from a focus on specific proteins and towards understanding the cellular abnormalities and processes that contribute to abnormal protein accumulation. Autophagy is the cell’s strategy to regulate metabolism by recycling intracellular materials such as proteins into their basic parts, so as to be re-utilized for other purposes [4]. This degenerative process can occur two ways, either dysfunctional proteins are captured by the lysosome for direct degradation [5], or dysregulated cytoplasmic materials are internalized into a double membrane structure referred to from here on out as “autophagosomes” before incorporating into the lysosome for degradation [6]. In addition to breaking down discrete cytosolic materials, autophagosomes can incorporate entire organelles for degradation; in fact, this often occurs to mitochondria in metabolically stressed cells [7].

The field has recently begun focusing on lysosomal and mitochondrial dysregulation as an early pathogenic “trigger” for AD [8]. In order for disease to manifest, cells must communicate their pathogenic substances, which includes proteins, signaling molecules, or genetic material to ensue disease propagation. This may be accomplished by packaging the pathogenic substances into compartments, known as extracellular vesicles (EVs), which travel between cells. Small EVs produced via endocytic pathways are released by nearly all cell types, including neurons [9]. EVs contain proteins, lipids, and genetic material such as DNA, mRNA, and microRNA. They are released into extracellular space to facilitate intercellular communication, export waste, and interact with the microglia [10, 11]. EVs are implicated in the spread of pathological proteins involved in neurodegenerative diseases including AD [12]. Over the last several years, EVs have emerged as a potential biomarker, potentiator, and therapeutic option, and this review seeks to synthesize the evidence collected to date. The goal of this article is to show that through the abnormal induction of autophagy seen in AD, the EV can serve in all three of these roles.

### Extracellular vesicles are an alternate end-product of endocytosis and interact with autophagic processes

#### Genesis of the extracellular vesicle

Small EVs are often cited having a diameter less than 150 nm [13, 14] and are responsible for the export of waste, interaction with the immune system, and communication between cells [10, 11, 13]. They are a byproduct of the endocytic pathway, and their release is contingent on the association of a multivesicular body (MVB) with the plasma membrane, which causes them to be released into the extracellular space [15]. The process of endocytosis is responsible for the internalization of extracellular proteins and biological material into the cell via invagination of the plasma membrane, through clathrin or non-clathrin mediated processes [16]. Endocytosed cargo is transported to a collection of other internalized bilayer micelles located near the periphery, and along with products of the trans-Golgi pathway create the MVB, mediated early on in the process by the key protein Rab5 [13]. Multiple tagging proteins such as the endosomal sorting complexes required for transport (ESCRT) complex are essential in determining the fate of MVBs and include the protein Vps4, which is involved with terminating the budding into the MVB [17]. Often, the MVBs are brought to the trans-Golgi network for refinement and modification of their cargo [18, 19] or for association with the autophagic processes for degradation and reutilization [20]. A key regulator in the process of autophagy is the interplay between insulin and the mammalian target of Rapamycin (mTOR) signaling [21–24].

Signaling from the mTOR pathway activates downstream effectors that stimulate cell growth, survival, cytoskeletal reorganization, and metabolic processes [22]. When these signals are turned off, such as in states of insulin resistance, the mTOR pathway ceases to initiate these proliferative cellular responses and, consequently, the cell switches on autophagy [25]. Therefore, it is no surprise that mTOR is a key modulator of aging and age-related disease and its initiation of autophagy has been linked to the accumulation of protein aggregates and dysfunctional organelles associated with cellular dysfunction. In fact, mTOR activity has been linked to disease progression in mouse models of AD [26, 27] and frontotemporal lobar dementia [28]. Finally, mTOR activity significantly impacts spatial learning and memory in aged mice [29, 30]. There is evidence suggesting that dysregulated insulin signaling [31], which is upstream of mTOR signaling, may contribute to AD pathogenesis. A recent study observed that upregulation of mTOR activity in vitro increased the levels of cytosolic tau, facilitated intracellular tau deposition and mediated tau localization to EVs [32]. Given that tau and Aβ are inducers of excitotoxicity in neurons [33], this provides evidence for the involvement of EVs as a potential feed forward loop, poising the system for pathogenesis.

### AD pathogenic proteins interact with endosomes and are found in EVs

Aβ and hyperphosphorylated tau also interact with the endocytic pathway and may contribute to EV biogenesis in AD. Secretion of EVs is generally thought to increase in response to stress or pathological conditions [14]. For example, the total number of circulating EVs were significantly increased in mice after chronic alcohol feeding and in humans suffering with alcoholism. [34]. In addition, increased EV release has been demonstrated in hypoxia [35], cisplatin- or irradiation-induced DNA damage [36, 37] and through oxidative stress [38]. Studies have shown that depolarization of cells, either by Ca2+ or K+ influx has been shown to increase MVB association with the plasma membrane, thereby increasing EV secretion [39, 40]. A recent study reports no difference in the total number and size distribution of EVs from the neuron isolated from blood between age-matched controls and mild cognitively impaired (MCI) subjects [41]. Inhibiting the formation, secretion, or uptake of EVs reduces the spread of oligomers and neurotoxicity [42]. Although additional studies are warranted, this suggests that the pathogenic cargo within the EVs are more important than the total number of EVs in AD. Below is a summary of proteins currently known to be related to neuronally derived EVs (NDEVs) in AD patients.

#### Aβ

The amyloidosis hypothesis of AD suggests that Aβ plaque accumulation leads to impaired neuronal signaling and eventually cell death [43]. Mutations in genes associated with this toxicity include aberrant activity of BACE 1, which then leads to accumulation of Aβ42 toxic plaques [44]. Aβ is a c-terminal cleavage product of the amyloid precursor protein (APP), which is a transmembrane protein found in endosomal membranes [45] and, to a lesser extent, the mitochondrial membrane [46]. Notably, APP has been shown to impede the functioning of the mitochondria in association with p53 and an increase of cytosolic reactive oxygen species, indicating that high levels of APP can lead to toxicity of the neuron [47]. There are multiple reports of these proteins and their substrates within EVs of in vitro AD models and EVs derived from neurons of AD patients. EVs isolated from neuronal cell lines show that inducing AD mutations can increase soluble APP (sAPP) protein β, sAPPα [13] and soluble Aβ1–42 [48]. Cells expressing AD-related genotypes have also shown an upregulation in C-end terminal fragments (a byproduct of APP after beta-secretase processing) [49], beta-secretase in released EVs, and co-localization of beta-secretase enzyme 1 with early EV markers [13]. Experiments involving depleting the media show that Aβ is associated with the EVs during the excretion process [50].

NDEVs isolated from AD patients also have a significant increase in soluble Aβ1–42 [41, 51–55]. Notably, NDEVs isolated from AD patients have increased levels of C-terminal fragments of the APP as well [45, 49]; compared to control subjects, NDEVs isolated from AD patients show enrichment of undigested lysosomal APP C-terminal fragments [45]. Aβ plaques also display interactive prion protein receptors, which is reported to increase the pathogenicity of the disease [56]. Recently muskelin, a protein involved with the reorganization of the cytoskeleton, has been implicated in the decision for either lysosomal degradation or EV secretion of the prion receptor protein, which may have implications in amyloidosis [57]. Finally, in a recent study between healthy controls and age-matched clinical AD cohorts, EV-bound Aβ strongly correlated with PET imaging of brain amyloid plaque load while unbound freely circulating Aβ did not [42]. Collectively, EV transfer of Aβ seems to be centrally involved in AD and can serve as a useful antemortem biomarker of disease progression.

#### Hyperphosphorylated tau

The gradual deposition of hyperphosphorylated tau protein within select neuronal types is central to the tauopathy component of AD [58]. For neuronal synapse formation to occur, microtubule elongation needs to occur. This is a process reliant on the incorporation of the neuronal microtubule-associated protein tau [59]. Tau also plays a role in axonal transport and neurite outgrowth, and all these functions are modulated by site-specific phosphorylation. Abnormal hyper-phosphorylation of tau leads to destabilization of microtubule networks, disruption of axonal transport processes and eventually to the accumulation of intra-neuronal neurofibrillary tangles, which are the other classical, pathological hallmarks of advanced stage AD [60, 61]. Despite the heterogeneity of onset in the disease, the progressive accumulation of neurofibrillary tangles are found to be highly correlative to the symptomatic onset of dementia in patients suffering with AD [62]. As tau becomes hyper-phosphorylated in neurons, cellular clearance machinery takes it up for degradation [63].

NDEVs from AD patients show an increase in tau phosphorylation at threonine 181 (p-T181-tau) and serine 396 (p-S396-tau) by 3–20-fold compared to NDEVs obtained from age-matched controls [41, 51, 52, 55]. Moreover, p-T181-tau levels are significantly higher in NDEVs isolated from later stage AD patients than from when they were still diagnosed with MCI [41], implicating either a disruption in the clearance capabilities or an enhancement in pathogenicity of EVs in later disease states. p-T181- and p-S396-tau were significantly lower in NDEVs of patients 1–10 years prior to their AD diagnosis [52].

#### Dysfunctional insulin signaling

**Fig. 1:**
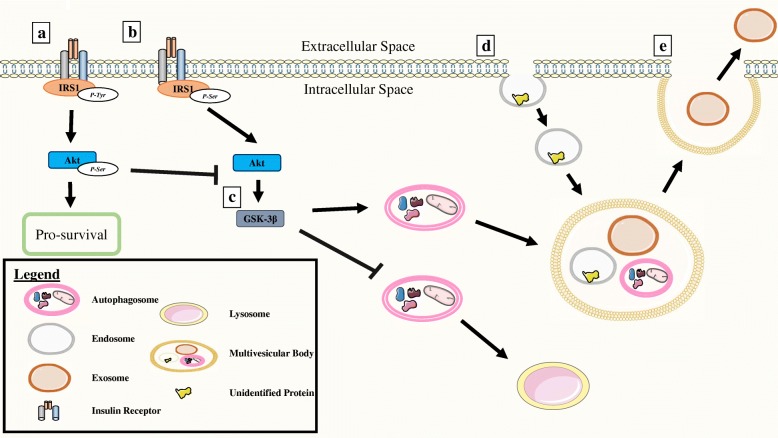
Outline for exosome secretion. **a** Normal stimulation of the insulin receptor leads to tyrosine phosphorylation of the insulin receptor susbtrate-1 (IRS1), initiating phosphorylated protein kinase b (Akt) inhibition of glycogen synthase kinase (GSK)-3β and activation of pro-survival signals. **b** Lack of stimulation or overstimulation at the insulin receptor leads to serine phosphorylation of the IRS1, which leads to dephosphorylated Akt activation of GSK-3β. **c** GSK-3β inhibits the autophagosome induction to the lysosome and promotes the introduction of the autophagosome to the multivesicular body (MVB). **d** Endocytosis allows for introduction of internalized proteins into the MVB. **e** MVB associates with the plasma membrane, allowing for exosome release from the cell

Central nervous system dysregulated insulin and peripheral hyperinsulinemia has been shown to be another highly associated phenomenon with AD [31, 64–66]. Insulin dysregulation can be characterized by low ratios of tyrosine phosphorylated insulin receptor substrate 1 (IRS1) to serine phosphorylated IRS1 [67, 68] and has been correlated with greater brain atrophy in humans with AD [55]. Chronic high-fat diet fed mice exhibit this post-translational modification shift in hippocampal slices [69]. Downstream, insulin signaling induces transduction pathways involving protein kinase B (Akt), and this dysregulated phosphorylation of IRS1 induces mTOR’s initiation of autophagy via de-phosphorylated Akt (Fig. 1). NDEVs isolated from AD patients have shown an increase in serine phosphorylation of IRS1 [70]. The group report that the differences in the IRS1 profiles were identifiable up to 10 years prior to clinical onset of AD. This suggests that proteins within NDEVs involved in insulin dysregulation may be a useful biomarker.

#### Synaptic proteins

The consequence of AD is a loss of neuronal health and function. NDEV cargo of AD patients display reduced levels of synapse proteins, including synaptotagmins, synaptophysin, synaptobrevin, synaptopodin, Rab3A, growth associated protein (GAP) 43, and neurogranin [71]. Also, low-density lipoprotein receptor-related protein (LRP) 6, heat shock factor protein 1, heat shock protein (HSP), and RE1 silencing transcription factor (REST 1) are also lower in NDEVs of AD patients [41, 51, 72]. The α-amino-3-hydroxy-5-methyl-4-isoxazolepropionic acid receptor (AMPAR) is also shown to be downregulated in NDEVs of AD patients. Additionally, neurexin 2α, GluA4-containing glutamate receptor, and neuroligin 1, all proteins essential for long-term potentiation processes, are all significantly lower in NDEVs of patients 6–11 years prior to AD diagnosis and, along with neuronal pentraxin 2, are all downregulated in NDEVs of AD patients [73]. These proteins are all involved with normal homeostatic processes of neurons. Further research into these cargo levels could be beneficial to clinicians who are currently searching for earlier biomarkers of disease; biomolecules shown to have changed concentrations in AD patient NDEVs are listed in Additional file 1: Table S1.

### EVs in biomedical research

#### EVs in toxicity studies

For AD propagation to occur via EVs, EVs must have the ability to transfer pathology between individual cells. Studies involving in vitro methods have supported the notion of pathogenic propagation by EVs. Eitan et al. used multiple genetically manipulated cell lines to characterize the cargo released in EVs and showed an increase in Aβ42 / Aβ40 ratio compared to controls [50]. They went on to show that co-incubation of rat cortical neurons with EVs isolated from AD transgenic cell lines had a similarly detrimental effect as co-incubation with medium concentrated in Aβ alone [48]. Co-incubation of neuronal cells derived from transgenic AD mouse models and EVs derived from adipose differentiated stem cells reduces pathology and apoptosis markers [74]. EVs isolated from *post-mortem* AD brains that contained increased levels of Aβ oligomers have been shown to act as toxic species to cultured neurons [75]. Blocking the formation, secretion or uptake of EVs was found to reduce both the spread of Aβ and the related cellular toxicity in these conditions [42]. These in vitro experiments provide sound mechanistic evidence of the pathogenicity potential of EVs but lack the in vivo support.

Probably the most compelling evidence for pathogenic spread is through a recent study by Winston and colleagues [41]. NDEVs isolated from patients diagnosed with either MCI or a more advanced stage of AD were microinjected into the hippocampus of wild type C57/BL6 mice. Phosphorylated tau reactivity increased in the mice one-month post-injection with MCI and AD derived EVs. Overall, this data demonstrates that NDEVs isolated from MCI and AD patients are capable of propagating tau pathology in normal mice. Additional studies have provided further evidence of this phenomenon with tau and other proteopathies, adding validity to the approach [76, 77]. Recently, when EV formation was blocked by inhibition of neutral sphingomyelinase-2 (nSMase2), AD pathology was decreased and improvements in memory were observed in an AD mouse model [78]. Altogether, the results of these provide rationale to pursue a means of inhibiting EV secretion as a potential therapy for individuals at risk for developing AD. Overall, these studies suggest a need for further investigation of the in vivo pathogenic potential of EVs.

The EV has the capability of transmitting disease through prion receptor protein (PrP) activity [79]. PrP is a cell surfaced anchored protein with unknown physiological functioning, but is highly associated with AD pathology [80]. Its pathological, misfolded form is protease-k-resistant and is implicated in encephalopathies [81]. Research in animal models of AD have shown that the prion receptor is necessary for the cognitive impairment associated with Aβ [82], however in other studies, PrP has been shown to mediate toxicity both in vitro and in vivo [83]. It is clear that this warrants further exploration, as the prion receptor likely to plays multiple roles associated with AD pathological proteins [84]. In AD, there is growing evidence for the prion receptor-containing EV being capable of spreading pathology [79, 85]. Aberrant autophagy may play a role in this spread [8]. While there is clearly a need for more research in order to understand this relationship, these findings suggest a potential mechanism connecting AD pathogenesis, the EV, and autophagy.

#### EVs as novel AD therapeutics

While EVs may play a role in the spreading of the disease, some studies have demonstrated a positive effect of introducing non-pathogenic EVs to alter the course of pathology and disease. This therapeutic effect was observed when EVs from young mice were found to significantly decrease aging-associated signaling molecules such as mTOR in aged mice [86]. Furthermore, EVs introduced into the brain of an AD transgenic mouse can benefit the clearance of toxic oligomers in vivo [87, 88], indicating the role of EVs in the interaction with amyloid plaques, which are known to have prion receptor proteins [13, 89]. As described above, Yuyama and colleagues showed that the introduction of naïve EVs into the brain of AD transgenic mouse models helped in the clearance of toxic fibrils [88, 90]. Others have suggested that EVs derived from mesenchymal stromal cells may have a therapeutic benefit in the promotion of neurovascular plasticity in other neurodegenerative diseases such as stroke in vivo [91]. Introducing exogenous EVs into the central nervous system is a potentially novel strategy for creating therapies for AD due to their ability to cross the blood-brain barrier efficiently [92] and their innate secretion of enzymes that are effective at breaking down toxic fibrils [93]. Furthermore, EVs derived from fibroblasts have been shown to induce axonal regeneration in an optic nerve injury model via wingless/integrated (Wnt) and mTOR signaling [94]. Additionally, EVs isolated from murine neuroblastoma (Neuro-2a) cells were able to reduce synaptic dysfunction and ameliorate Aβ pathology in a microglial dependent manner following intracerebral administration into APP heterozygotic transgenic mice expressing the Swedish and Indiana mutations [90]. The group demonstrated that EVs are incorporated into murine microglia in a glycosphingolipid (GSL)-glycan independent manner [90]. The field of EV research in the central nervous system is still in its infancy, and yet there are already promising therapeutic applications being brought forward.

## Conclusion

The research field has long held fast to proteopathic hypotheses of AD induction where cell to cell transfer of pathogenic proteins is postulated, and the topographical progression of neuritic plaques and neurofibrillary tangles have been extensively explored. Spreading through network connections could facilitate this propagation to distant areas within a neuronal network, possibly by a trans-synaptic mechanism [95]. The EV provides a suitable vector for spreading of pathogenic proteins within this context. More detailed studies are needed to prove that regional EVs transmit in patterns that mimic consortium to establish a registry for Alzheimer's disease (CERAD) and Braak staging schemata, however recent advancements in targeted EV labeling in vivo may provide a powerful tool for testing this hypothesis [96].

The pathogenic spreading of AD via cell-to-cell transfer remains far from being proven or universally accepted. Alternatively, the functional brain should also be understood as multiple interacting subsystems and connecting hubs that interact within a larger default network; all of which have been mapped using functional connectivity analyses. Brain imaging research has revealed that patients with AD have a specific anatomic pattern of reduced metabolism based on fMRI relative to age-matched controls [97]. The pattern of reduced metabolism bears a striking resemblance to the regions comprising the posterior components of the default network [98]. Intriguingly, the regions within the default network that show higher resting metabolism in healthy adults are also those that are most vulnerable to damage from AD. These findings lend to the notion that changes in the default network may somehow lead to or modify amyloid deposition [99]. These regional changes in the default mode may also differentially modify EV cargo in affected networks. However, quantifying changes in EV cargos and metabolism in the context of the default mode would be extremely difficult if not impossible given the lack of unique region specific markers for circulating EVs and the inaccessibility of specific brain tissue EVs from living patients.

Multiple studies have already characterized the vesicle in both genetically manipulated experimental situations and in blood taken from AD patients, bolstering its translatable reliability. Unfortunately, despite this growing evidence supporting the importance of EVs in AD, there is still a lack of standardization in methods [100]. Ultracentrifugation methods have been used to obtain highly pure EV fractions [101], but the reproducibility and efficiency of this method remains unclear. Many laboratories have instead opted to use microfiltration technologies, antibody-coated magnetic beads, microfluidic devices, or precipitation techniques using ExoQuick reagent, with the latter giving the most favorable outcomes in both quantity of EVs isolated and in quality of protein cargos [102]. Recently, the ExoQuick precipitation reagent was shown to yield comparable results to ultracentrifugation methods in independent studies of Down syndrome (DS) from independent labs [53, 103]. From blood, we consistently isolated 40% more NDEVs on average from DS blood when compared to controls based on the EV surface marker differentiation (CD) 81. Using ultracentrifugation methods, Levy and colleagues support this trend with evidence that *post-mortem* brain tissue isolated from individuals with DS expelled nearly 40% more EVs than non-DS controls based on levels of both Flotillin-1 and Flotillin-2 levels and acetylcholinesterase activity [103]. Thus it seems that orthogonal methods used by different laboratories can yield comparable outcomes in this context. While these data are promising, moving forward it is imperative that the field utilize a cohesive definition of the term “EV” in order to create inter-experimental reliability. A proposed characterization of EVs includes the combination of size profiling through particle analysis or electron microscopy and assessing the enrichment for EV-associated proteins such as CD 9, CD 63, CD 81, and/or apoptosis-linked gene 2-interacting protein X (Alix) [104–106].

There are interesting lines of research going into both the induction of AD using pathogenic EVs and the sequestration of toxic plaques using exogenous healthy EVs. Ultimately, it is the hope that using these gold standard approaches will provide reliable and reproducible insight into the role that the NDEV plays in pathogenesis of AD. Furthermore, there is a pressing need to determine the potential role of EVs derived from cell types other than neurons in AD pathophysiology. By ensuring concrete definitions of the EV, research can reliably characterize the role these vesicles collectively play in the development or potential treatment of AD. With the impending health care burden on our society, it is clear that the field needs an influx of novel approaches through which to study AD. The EV, with all of its connections to AD via aberrant metabolic pathways, provides a unique and promising research venture.

## Additional file

Additional file 1: **Table S1.** Proteins related to neuronally derived EVs of Alzheimer’s disease patients (DOCX 95 kb)

## Abbreviations

AD: Alzheimer’s disease
Akt: Protein kinase B
Alix: Apoptosis-linked gene 2-interacting protein X
AMPAR: α-amino-3-hydroxy-5-methyl-4-isoxazolepropionic acid receptor
APP: Amyloid precursor protein
Aβ: Beta-amyloid
BACE 1: Beta secretase enzyme 1
CD: Cluster of differentiation
CERAD: Consortium to establish a registry for Alzheimer's disease
DS: Down syndrome
ESCRT: Endosomal sorting complexes required for transport
EVs: Extracellular vesicles
GAP43: Growth-associated protein 43
HSP: Heat shock protein
IRS1: Insulin receptor substrate 1
LRP6: LDL receptor related protein
MCI: Mild cognitively impaired
mTOR: Mammalian target of rapamycin
MVB: Multivesicular body
NDEVs: Neuronally derived extracellular vesicles
Neuro-2a: Murine neuroblastoma
nSMase2: Neutral sphingomyelinase-2
PrP: Prion protein receptor
p-S396-tau: Tau phosphorylation at serine 396
p-T181-tau: Tau phosphorylation at threonine 181
REST: RE1-silencing transcription factor
sAPP: Soluble APP
Wnt: Wingless/integrated
