# Supplementary material for: Neuronally derived extracellular vesicles: an emerging tool for understanding Alzheimer’s disease
Source: Mol Neurodegener. 2019 Jun 10;14:22. doi: 10.1186/s13024-019-0317-5 (PMC6558712; doi:10.1186/s13024-019-0317-5)
Supplement: Supplementary file 1 — Table S1. Proteins related to neuronally derived EVs of Alzheimer’s disease patients (DOCX 95 kb) [file 13024_2019_317_MOESM1_ESM.docx]

| **Table 1. Proteins related to neuronally derived EVs of Alzheimer’s disease patients** | | | |  |
| --- | --- | --- | --- | --- |
| **Upregulated Proteins** | **Protein Function** | **AD Implication** | **EV Citations** | |
| Beta-amyloid (Aβ) | Neural growth and repair | Oligomerizes into plaques that are toxic | [1-11] | |
| Amyloid precursor protein (APP) | Regulates synapse formation/stability | Aβ made by APP accumulates as plaque in brain | [9, 12, 13] | |
| Tau phosphorylated at threonine 181 and serine 396 (p-T181-tau and p-S396-tau) | Signal transduction and axonal transport | Forms neurofibrillary tangles that are toxic | [1, 6, 7, 10] | |
| Cathepsin D | Localized in lysosomes cleaves diverse proteins | Accumulates into autolysosomes, elevated secretion associated with exosomes | [14] | |
|  |  |  |  | |
| Lysosome-associated membrane protein-1 (LAMP1) | Stabilizes polypeptide translocation systems into lysosomes | Protein degradation and recycling | [14] | |
| Serine 312 phosphorylated insulin receptor substrate-1 (IRS1) | Docking protein of the insulin receptor | Serine phosphorylation inhibits insulin signaling | [15, 16] | |
| Human E1/Ubiquitin-activating enzyme (E3/UBPL) | Protein quality control system | Protein degradation and recycling | [14] | |
| Pathogenic prion receptor protein (PrP) | Implicated in misfolded protein diseases | Mediates neurotoxicity of Aβ oligomers and NFTs | [8, 9, 17] | |
| **Downregulated Proteins** | **Protein Function** | **AD Implication** | **Citations** | |
| Heat shock protein 70 (HSP70), heat shock factor 1 | Protein chaperone | Inhibits protein aggregation and misfolding | [14, 18] | |
| Tyrosine phosphorylated insulin receptor substrate 1 (IRS1) | Docking protein of the insulin receptor | Tyrosine phosphorylation promotes insulin signaling | [15, 16] | |
| Synaptotagmins, synaptopodin, and synaptophysin | Involved in vesicular docking | Synapse loss leads to network dysfunctions | [19] | |
| Neurogranin | Facilitates synaptic transmission | Up regulated in CSF of AD patients | [6] | |
| Low density lipoprotein receptor related protein 6 (LRP6) | Upstream factor of REST transcription | Associated with APOE functions | [14, 18] | |
| RE1-silencing transcription factor (REST) | Enhance neuronal resistance to stresses and apoptosis in aging brains | Associated with aging and stress resistance | (39, 49, 70) | |
| Neurexin 2 alpha (NRXN2α); Neuroligin 1 (NLGN1); GluA4-containing glutamate receptor (AMPA4); neuronal pentraxin (NPTX2) | Essential excitatory synaptic proteins | Associated with amyloid beta oligomerization | [20] | |

**References**

1. Fiandaca MS, Kapogiannis D, Mapstone M, Boxer A, Eitan E, Schwartz JB, Abner EL, Petersen RC, Federoff HJ, Miller BL, Goetzl EJ: **Identification of preclinical Alzheimer's disease by a profile of pathogenic proteins in neurally derived blood exosomes: A case-control study.** *Alzheimer's & dementia : the journal of the Alzheimer's Association* 2015, **11:**600-607 e601.

2. Eitan E, Hutchison ER, Marosi K, Comotto J, Mustapic M, Nigam SM, Suire C, Maharana C, Jicha GA, Liu D, et al: **Extracellular Vesicle-Associated Abeta Mediates Trans-Neuronal Bioenergetic and Ca(2+)-Handling Deficits in Alzheimer's Disease Models.** *NPJ Aging Mech Dis* 2016, **2**.

3. Eitan E, Suire C, Zhang S, Mattson MP: **Impact of lysosome status on extracellular vesicle content and release.** *Ageing Res Rev* 2016, **32:**65-74.

4. Hamlett ED, Goetzl EJ, Ledreux A, Vasilevko V, Boger HA, LaRosa A, Clark D, Carroll SL, Carmona-Iragui M, Fortea J, et al: **Neuronal exosomes reveal Alzheimer's disease biomarkers in Down syndrome.** *Alzheimer's & dementia : the journal of the Alzheimer's Association* 2017, **13:**541-549.

5. Hamlett ED, Ledreux A, Potter H, Chial HJ, Patterson D, Espinosa JM, Bettcher BM, Granholm AC: **Exosomal biomarkers in Down syndrome and Alzheimer's disease.** *Free radical biology & medicine* 2018, **114:**110-121.

6. Winston CN, Goetzl EJ, Akers JC, Carter BS, Rockenstein EM, Galasko D, Masliah E, Rissman RA: **Prediction of conversion from mild cognitive impairment to dementia with neuronally derived blood exosome protein profile.** *Alzheimers Dement (Amst)* 2016, **3:**63-72.

7. Abner EL, Jicha GA, Shaw LM, Trojanowski JQ, Goetzl EJ: **Plasma neuronal exosomal levels of Alzheimer's disease biomarkers in normal aging.** *Annals of clinical and translational neurology* 2016, **3:**399-403.

8. Quek C, Hill AF: **The role of extracellular vesicles in neurodegenerative diseases.** *Biochemical and biophysical research communications* 2017, **483:**1178-1186.

9. Xiao T, Zhang W, Jiao B, Pan CZ, Liu X, Shen L: **The role of exosomes in the pathogenesis of Alzheimer' disease.** *Transl Neurodegener* 2017, **6:**3.

10. Polanco JC, Li C, Durisic N, Sullivan R, Gotz J: **Exosomes taken up by neurons hijack the endosomal pathway to spread to interconnected neurons.** *Acta neuropathologica communications* 2018, **6:**10.

11. Sardar Sinha M, Ansell-Schultz A, Civitelli L, Hildesjo C, Larsson M, Lannfelt L, Ingelsson M, Hallbeck M: **Alzheimer's disease pathology propagation by exosomes containing toxic amyloid-beta oligomers.** *Acta neuropathologica* 2018, **136:**41-56.

12. Laulagnier K, Javalet C, Hemming FJ, Chivet M, Lachenal G, Blot B, Chatellard C, Sadoul R: **Amyloid precursor protein products concentrate in a subset of exosomes specifically endocytosed by neurons.** *Cellular and molecular life sciences : CMLS* 2018, **75:**757-773.

13. Miranda AM, Lasiecka ZM, Xu Y, Neufeld J, Shahriar S, Simoes S, Chan RB, Oliveira TG, Small SA, Di Paolo G: **Neuronal lysosomal dysfunction releases exosomes harboring APP C-terminal fragments and unique lipid signatures.** *Nature communications* 2018, **9:**291.

14. Goetzl EJ, Boxer A, Schwartz JB, Abner EL, Petersen RC, Miller BL, Kapogiannis D: **Altered lysosomal proteins in neural-derived plasma exosomes in preclinical Alzheimer disease.** *Neurology* 2015, **85:**40-47.

15. Kapogiannis D, Boxer A, Schwartz JB, Abner EL, Biragyn A, Masharani U, Frassetto L, Petersen RC, Miller BL, Goetzl EJ: **Dysfunctionally phosphorylated type 1 insulin receptor substrate in neural-derived blood exosomes of preclinical Alzheimer's disease.** *FASEB journal : official publication of the Federation of American Societies for Experimental Biology* 2015, **29:**589-596.

16. Mullins RJ, Mustapic M, Goetzl EJ, Kapogiannis D: **Exosomal biomarkers of brain insulin resistance associated with regional atrophy in Alzheimer's disease.** *Hum Brain Mapp* 2017, **38:**1933-1940.

17. Heisler FF, Pechmann Y, Wieser I, Altmeppen HC, Veenendaal L, Muhia M, Schweizer M, Glatzel M, Krasemann S, Kneussel M: **Muskelin Coordinates PrP(C) Lysosome versus Exosome Targeting and Impacts Prion Disease Progression.** *Neuron* 2018, **99:**1155-1169 e1159.

18. Goetzl EJ, Boxer A, Schwartz JB, Abner EL, Petersen RC, Miller BL, Carlson OD, Mustapic M, Kapogiannis D: **Low neural exosomal levels of cellular survival factors in Alzheimer's disease.** *Annals of clinical and translational neurology* 2015, **2:**769-773.

19. Goetzl EJ, Kapogiannis D, Schwartz JB, Lobach IV, Goetzl L, Abner EL, Jicha GA, Karydas AM, Boxer A, Miller BL: **Decreased synaptic proteins in neuronal exosomes of frontotemporal dementia and Alzheimer's disease.** *FASEB journal : official publication of the Federation of American Societies for Experimental Biology* 2016, **30:**4141-4148.

20. Goetzl EJ, Abner EL, Jicha GA, Kapogiannis D, Schwartz JB: **Declining levels of functionally specialized synaptic proteins in plasma neuronal exosomes with progression of Alzheimer's disease.** *FASEB journal : official publication of the Federation of American Societies for Experimental Biology* 2018, **32:**888-893.
